# Microencapsulated IL-12 Drives Genital Tract Immune Responses to Intranasal Gonococcal Outer Membrane Vesicle Vaccine and Induces Resistance to Vaginal Infection with Diverse Strains of Neisseria gonorrhoeae

**DOI:** 10.1128/msphere.00388-22

**Published:** 2022-12-20

**Authors:** Yingru Liu, Laura A. Hammer, Jikke Daamen, Michiel Stork, Nejat K. Egilmez, Michael W. Russell

**Affiliations:** a Therapyx, Inc., Buffalo, New York, USA; b Intravacc, Bilthoven, The Netherlands; c University of Louisville, Louisville, Kentucky, USA; d University at Buffalo, Buffalo, New York, USA; University of Florida

**Keywords:** *Neisseria gonorrhoeae*, genital tract immunity, interleukin-12, intranasal, microencapsulation, outer membrane vesicles, vaccine

## Abstract

An experimental gonococcal vaccine consisting of outer membrane vesicles (OMVs) and microsphere (ms)-encapsulated interleukin-12 (IL-12 ms) induces Th1-driven immunity, with circulating and genital antibodies to Neisseria gonorrhoeae, after intravaginal (i.vag.) administration in female mice, and generates resistance to vaginal challenge infection. Because i.vag. administration is inapplicable to males and may not be acceptable to women, we determined whether intranasal (i.n.) administration would generate protective immunity against N. gonorrhoeae. Female and male mice were immunized i.n. with gonococcal OMVs plus IL-12 ms or blank microspheres (blank ms). Responses to i.n. immunization were similar to those with i.vag. immunization, with serum IgG, salivary IgA, and vaginal IgG and IgA antigonococcal antibodies induced when OMVs were administered with IL-12 ms. Male mice responded with serum IgG and salivary IgA antibodies similarly to female mice. Gamma interferon (IFN-γ) production by CD4^+^ T cells from iliac lymph nodes was elevated after i.n. or i.vag. immunization with OMVs plus IL-12 ms. Female mice immunized with OMVs plus IL-12 ms by either route resisted challenge with N. gonorrhoeae to an equal extent, and resistance generated by i.n. immunization extended to heterologous strains of N. gonorrhoeae. Detergent-extracted OMVs, which have diminished lipooligosaccharide, generated protective immunity to challenge similar to native OMVs. OMVs from mutant N. gonorrhoeae, in which genes for Rmp and LpxL1 were deleted to eliminate the induction of blocking antibodies against Rmp and diminish lipooligosaccharide endotoxicity, also generated resistance to challenge infection similar to wild-type OMVs when administered i.n. with IL-12 ms.

**IMPORTANCE** We previously demonstrated that female mice can be immunized intravaginally with gonococcal outer membrane vesicles (OMVs) plus microsphere (ms)-encapsulated interleukin-12 (IL-12 ms) to induce antigonococcal antibodies and resistance to genital tract challenge with live Neisseria gonorrhoeae. However, this route of vaccination may be impractical for human vaccine development and is inapplicable to males. Because intranasal immunization has previously been shown to induce antibody responses in both male and female genital tracts, we have evaluated this route of immunization with gonococcal OMVs plus IL-12 ms. In addition, we have refined the composition of gonococcal OMVs to reduce the endotoxicity of lipooligosaccharide and to eliminate the membrane protein Rmp, which induces countereffective blocking antibodies. The resulting vaccine may be more suitable for ultimate translation to human application against the sexually transmitted infection gonorrhea, which is becoming increasingly resistant to treatment with antibiotics.

## INTRODUCTION

Despite public health efforts, infection with Neisseria gonorrhoeae (i.e., gonorrhea) remains highly prevalent, with worldwide incidence currently estimated at 87 million new infections per year (1). In the United States, incidence increased by 66% over the years 2014 to 2018, exceeding 580,000 reported cases in 2018 (2), although the true incidence is believed to be even greater. N. gonorrhoeae is listed as an “urgent threat” among human pathogens because of the emergence of resistance to currently available antibiotics (3), and cases of extreme resistance have been reported. Although not usually lethal, gonococcal infection is a major cause of reproductive tract morbidity, especially in women, because ascending infection leads to inflammatory damage to the uterus and Fallopian tubes, resulting in tubal factor infertility, increased risk for ectopic pregnancy, and debilitating pelvic inflammatory disease (4). Infection during pregnancy can result in adverse outcomes, including miscarriage, and a neonate delivered through an infected birth canal is at risk of eye infection, which can cause blindness if not treated. While the search for effective new antibiotics continues, the track record of N. gonorrhoeae in rapidly acquiring resistance to each antibiotic deployed against it suggests that this approach might afford only a temporary solution and that another solution, like a vaccine, will be needed for long-term control of this infection. Previous efforts at vaccine development were unsuccessful (5–7), but recent advances in comprehending immunity to N. gonorrhoeae (8) coupled with the finding that immunization against the related organism Neisseria meningitidis might provide modest cross-protective immunity against N. gonorrhoeae (9) have reinvigorated efforts at gonococcal vaccine development.

We previously demonstrated that an experimental intravaginal (i.vag.) vaccine consisting of native gonococcal outer membrane vesicles (nOMVs), initially obtained by lithium acetate (LiAc) extraction (LiAcOMVs), and microsphere (ms)-encapsulated interleukin-12 (IL-12 ms) (10) induces Th1-driven immune responses and resistance to N. gonorrhoeae infection in mice (11). The i.vag. immunization regimen, although shown to be effective, is thought to be impracticable for human prophylactic application and is inapplicable for males. However, numerous studies have shown that intranasal (i.n.) immunization, in humans as well as in experimental animals, elicits responses in the genital tract in both males and females (12–19). A comparative study in mice demonstrated that i.n. immunization was more effective than i.vag. immunization for generating genital antibody responses (20), likely because the i.n route elicits responses in the common mucosal immune system through inductive sites located in the nasopharynx and thereby disseminates activated T and B cells to remote effector sites (21). In contrast, the genital tract lacks mucosal immune inductive site tissues akin to intestinal Peyer’s patches and therefore has a limited ability to mount immune responses and, especially, to disseminate them to remote effector sites (22). We therefore hypothesized that i.n. immunization with gonococcal OMVs plus IL-12 ms should also effectively induce antigonococcal antibodies in the genital tract as well as in serum and other secretions and generate protective immunity against vaginal N. gonorrhoeae infection.

An intriguing aspect of gonococcal immunity induced by means of OMVs plus IL-12 ms given i.vag. is that it is not strain specific but extends to additional strains represented by commonly used “laboratory” strains, including FA1090, MS11, and FA19, as well as a few uncharacterized clinical isolates (11). Having access to the WHO reference strains (23), we have now also included these well-characterized strains in our analysis. In addition, because OMVs contain endotoxic lipooligosaccharide (LOS), which is highly inflammatory and reactogenic, we have examined detergent-extracted OMVs (deOMVs), which have reduced LOS content. We compared i.n. immunization with deOMVs and nOMVs obtained by extraction with EDTA in a side-by-side experiment to evaluate the immune responses and protective potential against vaginal gonococcal infection. Detergent extraction, however, also partially removes other antigenic materials, which might render the OMVs less immunogenic and less able to induce protective immunity. A more precise way of reducing LOS toxicity is by means of genetic mutation: for example, by deleting *lpxL1* to eliminate one acyl group in lipid A (24). In addition, the gonococcal membrane protein Rmp induces blocking antibodies that inhibit complement-mediated bacteriolysis induced by antibodies to LOS or porin (25). Deletion of Rmp also enhances the yield of OMVs as the outer membrane is more loosely attached to bacterium (26). Therefore, OMVs prepared from an N. gonorrhoeae double mutant in which the genes *lpxL1* and *rmp* were deleted have also been evaluated as an i.n. vaccine that might ultimately be more acceptable for human application.

## RESULTS

### Comparison of i.n. and i.vag. immunization.

**FIG 1:**
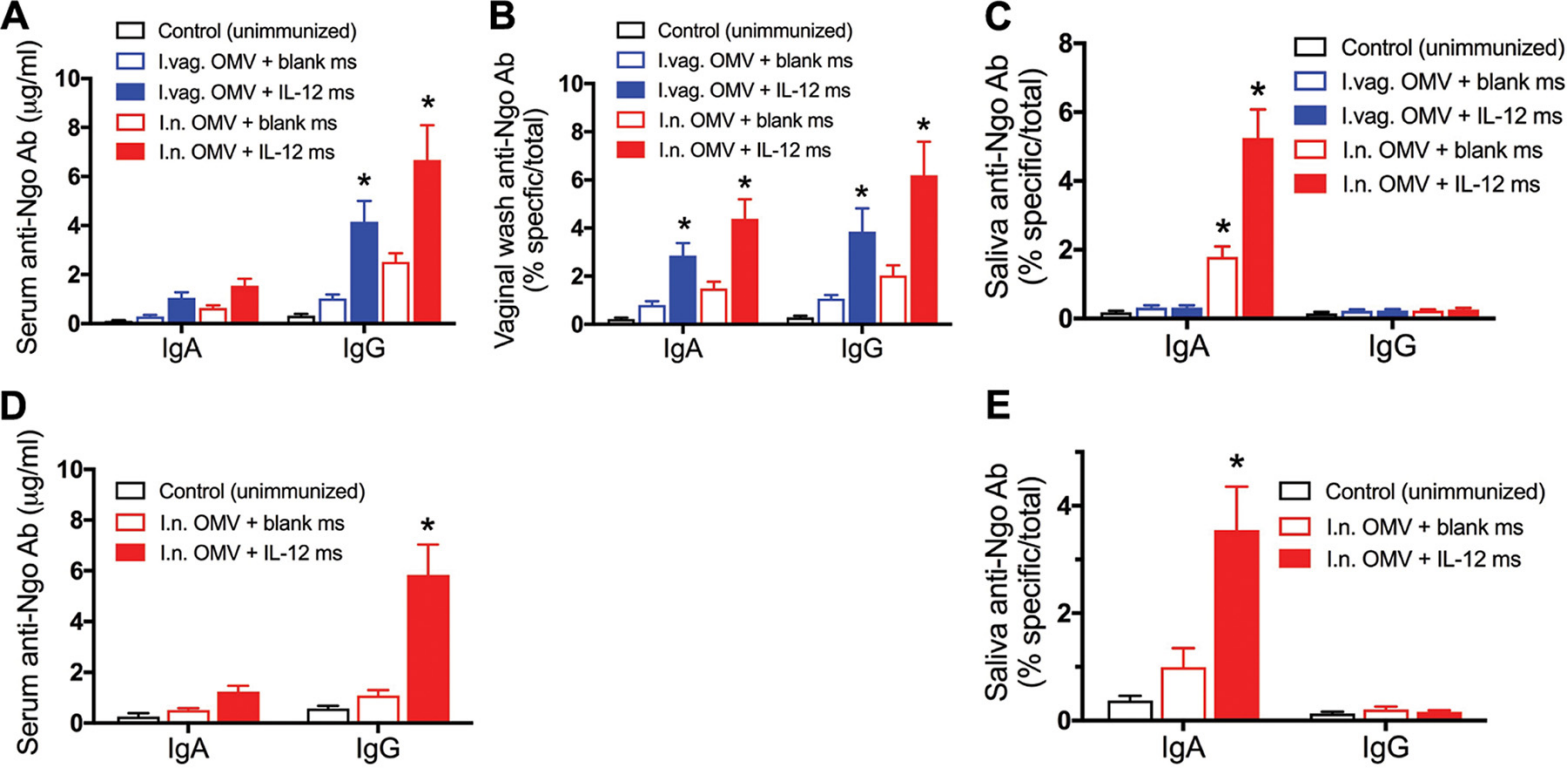
Comparison of antibody responses in mice immunized i.vag. or i.n. with gonococcal LiAcOMVs plus blank ms or IL-12 ms. (A) Serum IgG and IgA (female); (B) vaginal wash IgG and IgA (female); (C) salivary IgG and IgA (female); (D) serum IgG and IgA (male, i.n. immunized only); (E) salivary IgG and IgA (male, i.n. immunized only). Anti-Ngo Ab, antigonococcal antibody. Data are shown as means ± standard errors of the means (SEM) (*n* = 5). *, *P* < 0.01 relative to control (unimmunized) mice (2-way ANOVA with Tukey’s multiple comparisons).

Groups of female mice were immunized with LiAcOMVs (FA1090) and IL-12 ms given either i.n. or i.vag. in the same dose and on the same schedule: i.e., 30 μg of OMV protein plus either blank ms or ms containing 1 μg of IL-12 twice with a 2-week interval between doses. A control group was left unimmunized. Two weeks after the second immunization, samples of serum, vaginal wash, and saliva were collected from all mice and assayed for antigonococcal (FA1090) IgG and/or IgA antibodies by enzyme-linked immunosorbent assay (ELISA). Overall, mice immunized i.n. tended to show stronger antibody responses than those immunized i.vag., although the difference was not statistically significant (Fig. 1). Moreover, mice receiving IL-12 ms along with OMVs by either route also showed stronger responses than those given OMVs with blank ms as observed previously for i.vag. immunization (11). Serum IgG but not IgA antibodies were significantly elevated in mice immunized with OMVs plus IL-12 ms by either route (Fig. 1A). In vaginal wash, both IgG and IgA antibodies were developed (Fig. 1B), whereas in saliva, only IgA antibodies were detected and only in mice immunized i.n. (Fig. 1C). Mice immunized with OMVs plus blank ms showed minor elevation of serum IgG as well as vaginal IgG and IgA antibodies, but these were not significantly elevated compared to levels in unimmunized controls (Fig. 1A and B). However, female mice immunized i.n. with OMVs plus blank ms appeared to show significantly increased salivary IgA antibodies (Fig. 1C).

In parallel, groups of male mice were also immunized i.n. with OMVs plus either IL-12 ms or blank microspheres, and a third group was left unimmunized. Male mice responded to i.n. immunization with OMVs plus IL-12 ms with serum IgG (Fig. 1D) and salivary IgA (Fig. 1E) antigonococcal antibodies in essentially the same way as female mice immunized i.n. Male mice given OMVs plus blank ms did not show significantly elevated serum or salivary antibodies (Fig. 1D and E).

**FIG 2:**
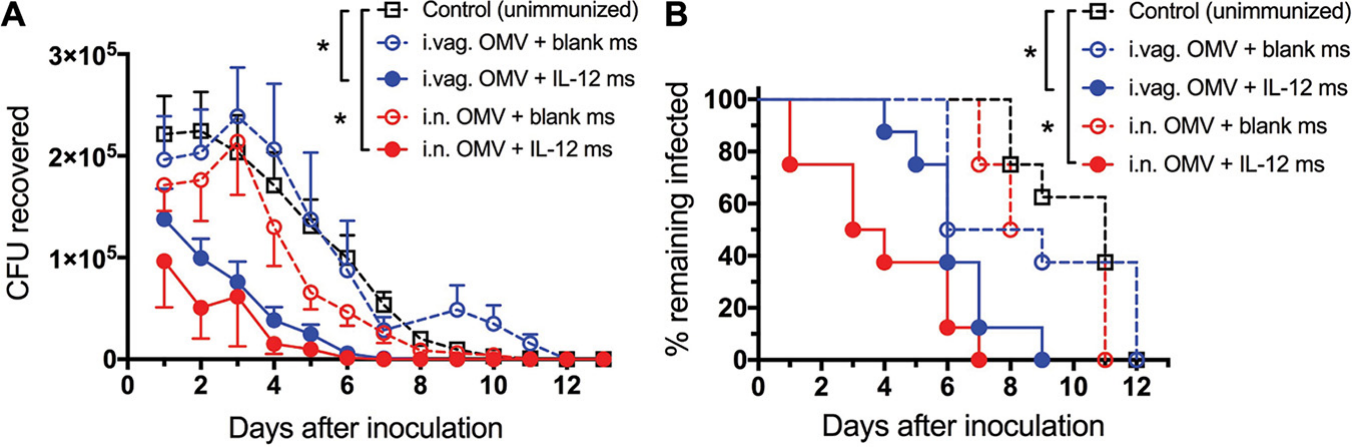
Time course of gonococcal infection after challenge of mice immunized i.n. or i.vag. with gonococcal LiAcOMVs plus blank ms or IL-12 ms with 5 × 10^6^ CFU of N. gonorrhoeae FA1090. (A) Recovery of N. gonorrhoeae on vaginal swabs at each time point. Data are shown as means ± SEM (*n* = 8 or 9 mice per group). *, *P* < 0.01 (2-way repeated measures ANOVA with Tukey’s multiple comparisons). (B) Percentage of mice remaining infected at each time point. *, *P* < 0.001 (Kaplan-Meier log rank test).

Three weeks after the second immunization, female mice were prepared for challenge with N. gonorrhoeae (FA1090) by administration of estradiol and antibiotics to quell overgrowth of commensals resulting from estradiol treatment, and the course of infection after inoculation was monitored daily by vaginal swabbing and quantitative plating for recovery of live N. gonorrhoeae cells (27). Mice immunized with OMVs plus blank ms by either i.n. or i.vag. routes cleared the infection at a similar rate to the unimmunized control mice (Fig. 2A), and all animals had cleared the infection by day 11 or 12 Fig. 2B). In contrast, mice immunized with OMVs plus IL-12 ms given i.n. or i.vag. cleared the infection significantly faster than the control mice (Fig. 2A), with all animals in each group clearing the infection by days 7 and 9, respectively (Fig. 2B).

**FIG 3:**
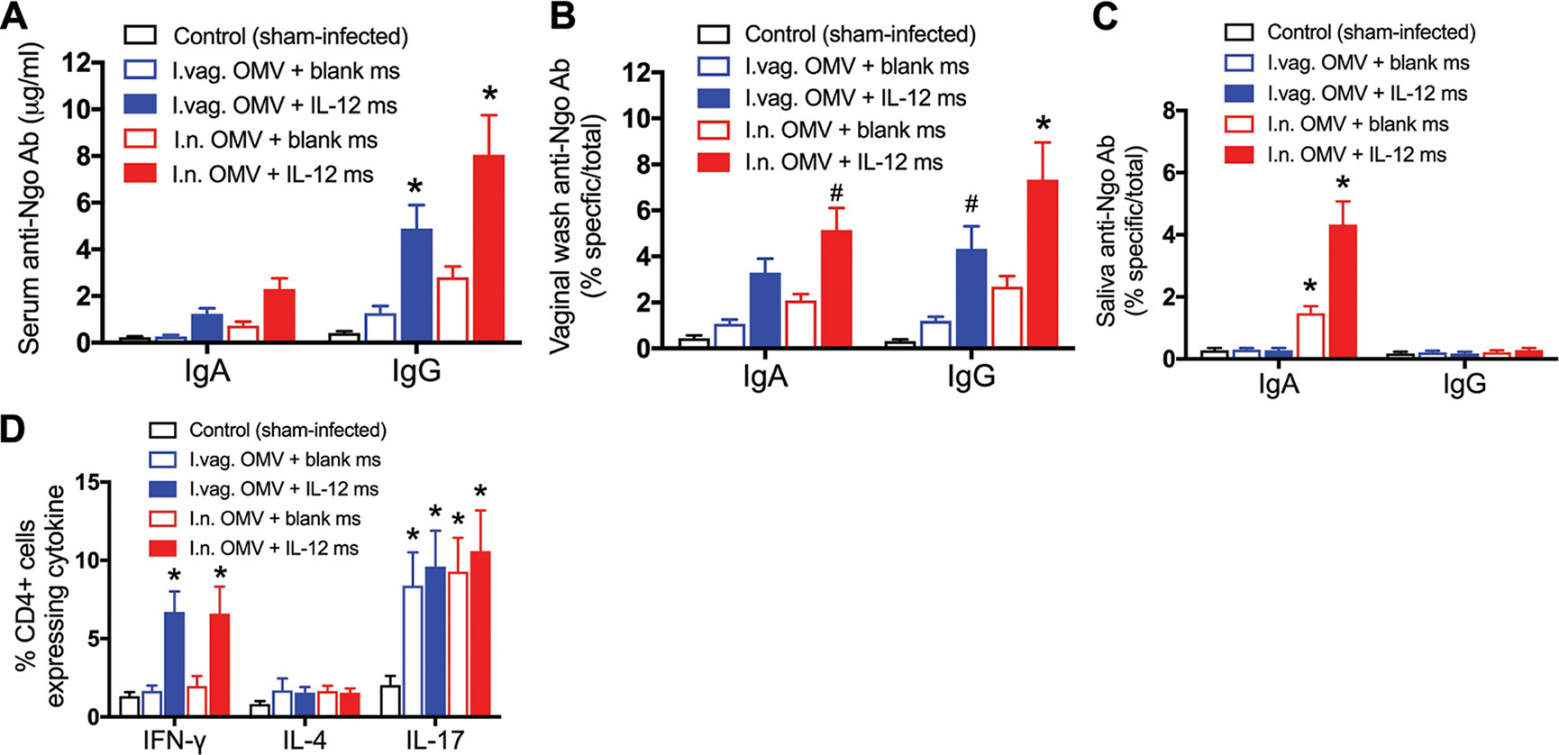
Immune responses in mice immunized i.n. or i.vag. with gonococcal LiAcOMVs plus blank ms or IL-12 ms after clearance of challenge infection with N. gonorrhoeae. (A to C) Antigonococcal antibodies in serum (A), vaginal wash (B), or saliva (C). ^#^, *P* < 0.05; *, *P* < 0.01 (2-way ANOVA with Tukey’s multiple comparisons). (D) CD4^+^ T cell cytokine responses in iliac lymph nodes. Data are shown as means ± SEM (*n* = 5). *, *P* < 0.01 comparing OMVs plus IL-12 ms and OMVs plus blank ms for each route of administration (Student's *t* test).

After clearance of the infection in all mice, ~2 weeks after challenge, mice were euthanized, samples of serum, saliva, and vaginal wash were collected, and the draining iliac lymph nodes (ILNs) were harvested. Antigonococcal antibody responses in serum, vaginal washes, and saliva were broadly similar to those assayed after immunization and before challenge (compare Fig. 1A to C and Fig. 3A to C). Cells from the ILNs were stained for intracellular cytokines (gamma interferon [IFN-γ], IL-4, and IL-17) and for surface CD4 and analyzed by flow cytometry. Mice immunized with OMVs plus IL-12 ms (but not blank ms) given by either the i.n. or i.vag. route developed CD4^+^ cells staining for intracellular IFN-γ (Fig. 3D), whereas none of the groups displayed CD4^+^ cells staining for IL-4, and all infected mice developed CD4^+^ IL-17^+^ cells regardless of immunization (Fig. 3D).

**FIG 4:**
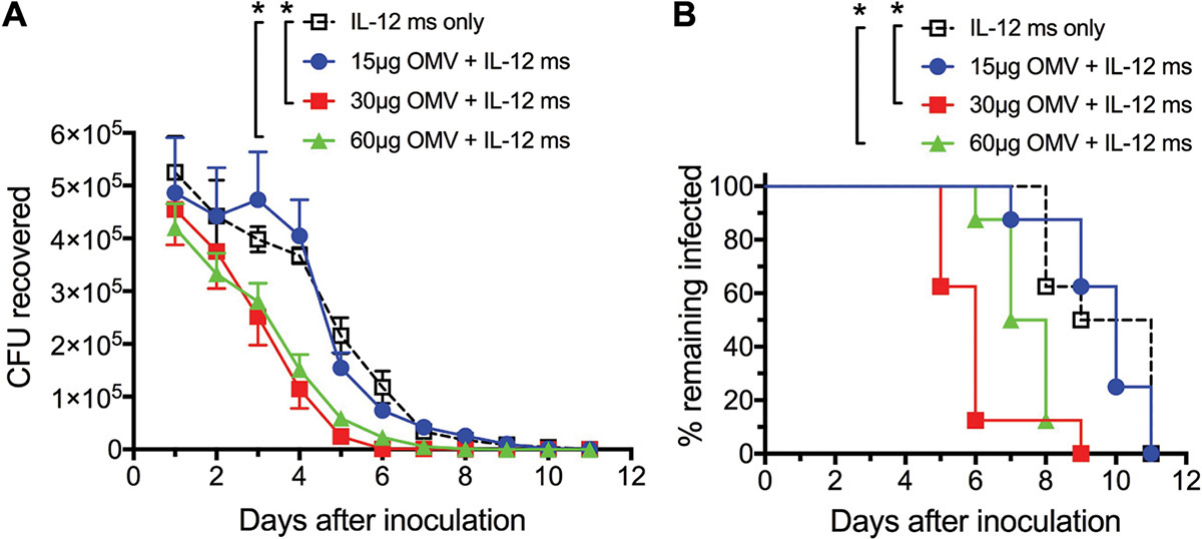
Minimum gonococcal OMV dose requirements for i.n. immunization with fixed IL-12 ms concentration. (A and B) Mice were immunized with 15, 30, or 60 μg FA1090 LiAcOMVs (protein) plus IL-12 ms or with IL-12 ms only for two doses with a 14-day interval and challenged 2 weeks later with N. gonorrhoeae FA19. (A) CFU recovered at each time point (means ± SEM; *n* = 9). *, *P* < 0.01 (2-way ANOVA with Tukey’s multiple comparisons). (B) Percentage of mice remaining infected at each time point. *, *P* < 0.01 (Kaplan-Meier log rank test).

To determine an optimal immunization dose of FA1090 LiAcOMVs by the i.n. route, groups of female mice were immunized i.n. with 15, 30, or 60 μg of OMVs (based on total protein) together with IL-12 ms (1 μg IL-12); control mice were given IL-12 ms alone, and immunizations were repeated after 14 days. When challenged 2 weeks later with N. gonorrhoeae FA19, mice immunized with 15 μg OMVs (plus IL-12 ms) cleared the infection no faster than mice given only IL-12 ms, whereas mice given 30 μg or 60 μg OMVs (plus IL-12 ms) cleared the infection significantly faster, with no difference between these two groups (Fig. 4A and B). Thus, 30 μg OMVs appeared to be a sufficient dose and was used for all subsequent experiments.

### Cross-reactivity against diverse strains.

In our previous studies on i.vag. immunization (11), we found that immunization of mice with LiAcOMVs from strain FA1090 induced resistance to challenge not only with the same strain but also with two other strains commonly used in laboratory studies, FA19 and MS11. Similar experiments using i.n. immunization with LiAcOMVs prepared from these two strains and administered with IL-12 ms revealed heterologous cross-protection against the other strains (see Fig. S1 in the supplemental material).

10.1128/msphere.00388-22.1 FIG S1 Cross-protection against heterologous strains of N. gonorrhoeae induced by i.n. immunization with various strains of gonococcal LiAcOMVs plus IL-12 ms. (A and B) Immunization with FA19 LiAcOMVs plus IL-12 ms (A) or blank ms (B), challenged with MS11 (*n* = 9). *, *P* = 0.0230 (Kaplan-Meier log rank test). (C and D) Immunization with MS11 LiAcOMVs plus IL-12 ms (C) or blank ms (D), challenged with FA1090 (*n* = 8 or 9). *, *P* < 0.01 (Kaplan-Meier log rank test). (E and F) Immunization with MS11 LiAcOMVs plus IL-12 ms (E) or blank ms (F), challenged with FA19 (*n* = 9). *, *P* = 0.0120 (Kaplan-Meier log rank test). (G and H) Immunization with FA19 LiAcOMV plus IL-12 ms (G) or blank ms (H), challenged with FA1090 (*n* = 9). *, *P* < 0.02 (2-way ANOVA). Download FIG S1, PDF file, 1.2 MB. Copyright © 2022 Liu et al. 2022 Liu et al. https://creativecommons.org/licenses/by/4.0/ This content is distributed under the terms of the Creative Commons Attribution 4.0 International license.

To further evaluate cross-protection against a wider range of gonococcal strains, we first performed a screening ELISA for IgG antibodies in pooled serum from mice immunized i.n. with OMVs from strain FA1090 or FA19 (plus IL-12 ms) against the panel of 16 WHO reference strains of N. gonorrhoeae (23) in comparison to the homologous immunizing strains. IgG antibodies in both serum pools cross-reacted with the WHO strains to various extents, ranging from 23% to 71% relative to the homologous immunizing strain (Table S1). Although strains FA1090 and FA19 express different PorB types (1b and 1a, respectively), there was no discernible relationship between cross-reactivity and the PorB type of the WHO strains: both pools of serum showed various levels of IgG antibodies against the WHO strains regardless of PorB type.

10.1128/msphere.00388-22.2 TABLE S1 Cross-reactivity of sera from mice immunized i.n. with gonococcal OMVs against N. gonorrhoeae WHO reference strains. Results are shown as the percentage of IgG reactivity against each strain relative to reactivity against the homologous immunizing strain (shown as 100%). Download Table S1, PDF file, 0.02 MB. Copyright © 2022 Liu et al. 2022 Liu et al. https://creativecommons.org/licenses/by/4.0/ This content is distributed under the terms of the Creative Commons Attribution 4.0 International license.

To determine whether i.n. immunization with OMVs from strain FA1090 could induce resistance to infection with any WHO strains, groups of 8 female mice were immunized i.n. with FA1090 OMVs (30 μg protein) plus either blank ms or IL-12 ms (1 μg IL-12) twice with a 2-week interval. Three weeks later, the mice were inoculated vaginally with N. gonorrhoeae WHO strain F, L, or W (which had been rendered streptomycin resistant) or with FA1090 (which is naturally streptomycin resistant), and the course of infection was monitored daily by vaginal swabbing and plating.

**FIG 5:**
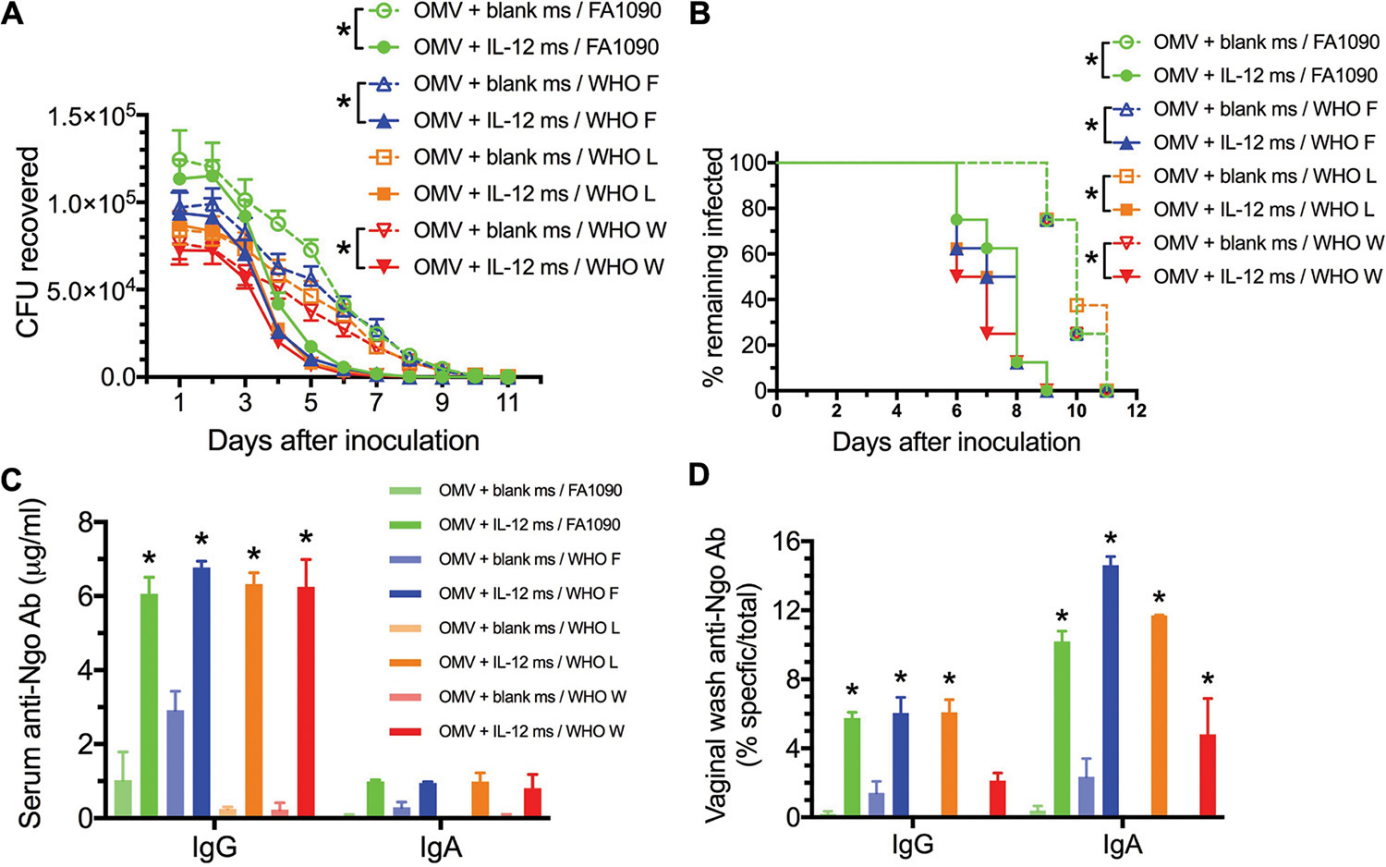
Mice immunized i.n. with FA1090 LiAcOMVs plus IL-12 ms show resistance to genital infection with WHO reference strains similar to FA1090. (A) Recovery of N. gonorrhoeae on vaginal swabs at each time point. Data are shown as means ± SEM (*n* = 8). *, *P* < 0.05 (2-way ANOVA with Sidak’s multiple paired comparisons). (B) Percentage of mice remaining infected at each time point. *, *P* < 0.001 (Kaplan-Meier log rank test). (C and D) serum (C) and vaginal wash (D) antigonococcal antibody levels in samples collected after clearance of infection, assayed against each challenge strain as shown. Data are shown as means ± SEM of results from pooled samples (*n* = 5) from each group. *, *P* < 0.01 (2-way ANOVA with Tukey’s multiple comparisons) relative to the corresponding group immunized with FA1090 OMVs plus blank ms.

The WHO strains were cleared from mice immunized i.n. with FA1090 OMVs plus IL-12 ms at broadly similar rates to the clearance of FA1090 (Fig. 5). Two-way analysis of variance (ANOVA) with Sidak multiple comparisons showed significant reductions in gonococci recovered between groups immunized with OMVs plus IL-12 ms or OMVs with blank ms for the strains FA1090, WHO F, and WHO W (Fig. 5A). Kaplan-Meier analysis of clearance times showed significantly faster clearance for all strains in mice immunized with FA1090 OMVs plus IL-12 (median clearance by days 8, 7.5, 7.5, and 6.5 for FA1090 and WHO strains F, L, and W, respectively) than in mice immunized with FA1090 OMVs plus blank ms (median clearance by day 10 for all strains) (Fig. 5B). After clearance of the infections, at day 14 after inoculation, mice were euthanized and samples of serum and vaginal wash were collected for antibody assay. Mice immunized with FA1090 OMVs plus IL-12 ms demonstrated serum IgG antibodies against the corresponding challenge strains at significantly higher levels than mice immunized with FA1090 OMVs plus blank ms (Fig. 5C). Serum IgA antibodies were not significantly elevated (Fig. 5C). Vaginal IgA antibodies against the corresponding challenge strains were also elevated in mice immunized with FA1090 OMVs plus IL-12 ms (Fig. 5D), and IgG antibodies were significantly elevated against three of the four strains (Fig. 5D). Mice immunized with FA1090 OMVs plus blank ms did not generate significant vaginal IgA or IgG antibodies against the gonococcal strains used (Fig. 5D).

### Detergent-extracted OMVs.

To obviate potential problems arising from the endotoxic properties of LOS, which is present in OMVs, we first tested the immunogenicity and protective potential of a vaccine based on detergent-extracted OMV (deOMVs), which have diminished LOS content in comparison with native OMVs (nOMVs). Groups of 8 female mice were immunized i.n. with either deOMVs or EDTA-extracted nOMVs derived from N. gonorrhoeae strain FA19 (30 μg based on total protein), each together with either blank ms or IL-12 ms (1 μg IL-12) twice with a 12-day interval between doses. A fifth control group was sham immunized with vehicle only. Two weeks later, all mice were challenged with N. gonorrhoeae strain FA1090, and the course of infection was monitored by vaginal swabbing and plating for recovery of N. gonorrhoeae. At termination after clearance of the infection (14 days after inoculation), samples of serum and vaginal wash were collected for antibody assay, and iliac lymph nodes were harvested for analysis of cytokine production by CD4^+^ T cells.

**FIG 6:**
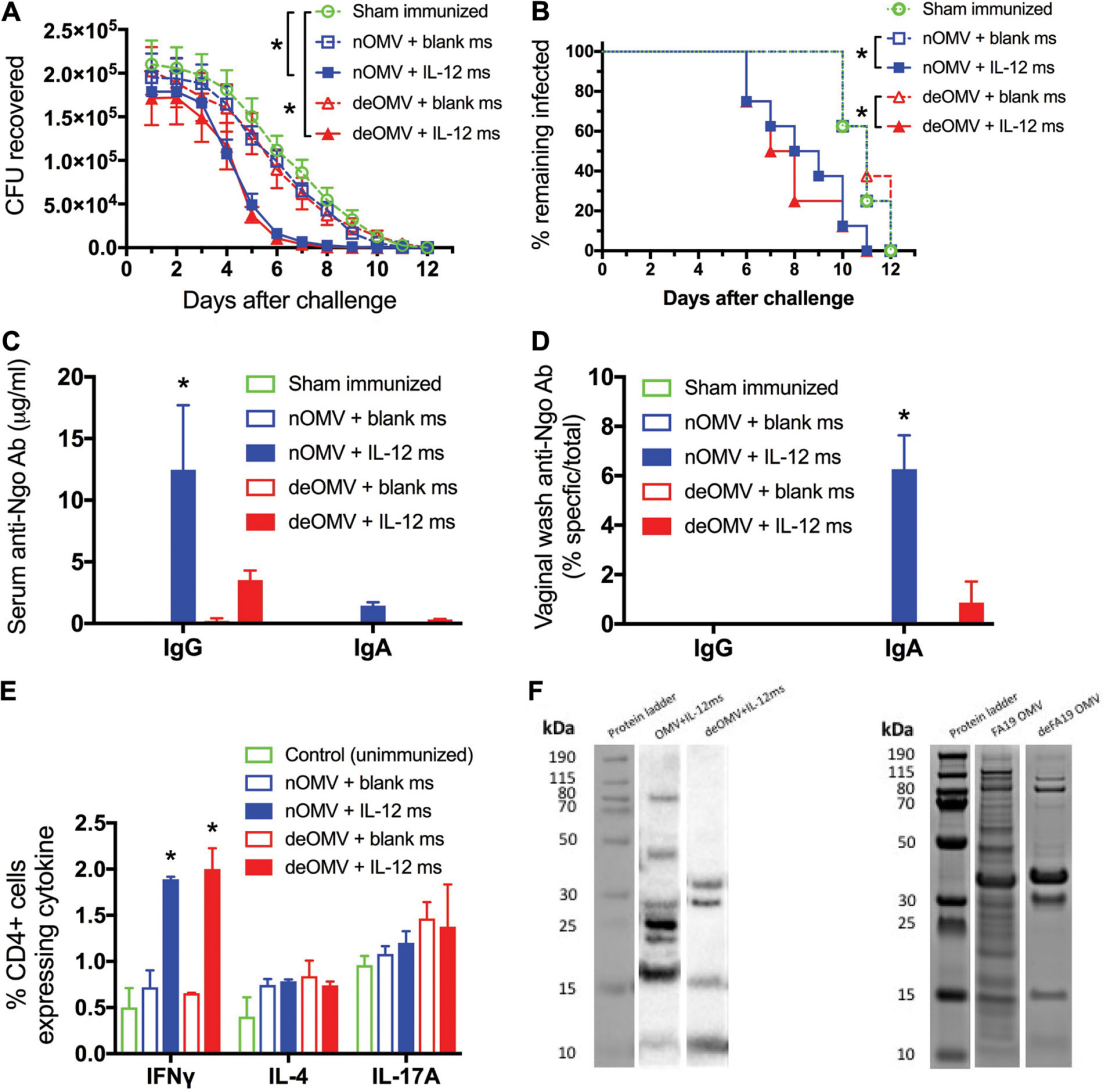
Comparison of i.n. immunization with native (nOMVs) and detergent-extracted (deOMVs) FA19 OMVs. Mice were immunized i.n. on days 0 and 12 with nOMVs or deOMVs (30 μg protein) plus IL-12 ms or blank ms and challenged 2 weeks later with 5 × 10^6^ CFU of N. gonorrhoeae FA1090. (A) Recovery of gonococci (mean ± SEM; *n* = 8) on vaginal swabs. *, *P* < 0.02 (2-way ANOVA with Tukey’s multiple comparisons). (B) Percentage of mice remaining infected at each time point. *, *P* < 0.01 (Kaplan-Meier log rank test). (C and D) Serum antibodies (C) and vaginal wash antibodies (D) in pooled group samples collected at termination. *, *P* < 0.01 (2-way ANOVA with Tukey’s multiple comparisons) relative to the corresponding group immunized with OMVs plus blank ms. (E) intracellular cytokine staining in CD4^+^ T cells from iliac lymph nodes at termination. *, *P* < 0.001 comparing OMVs plus IL-12 ms versus OMVs plus blank ms for both nOMVs and deOMVs (Student's *t* test). (F) Western blot analysis of IgG antibodies in pooled sera from mice immunized with nOMVs or deOMVs plus IL-12 ms (left lanes) compared to Coomassie blue-stained profile of proteins in nOMVs and deOMVs separated by SDS-PAGE (right lanes).

Mice immunized with either deOMVs or nOMVs plus IL-12 ms resisted challenge to a similar extent (median clearance by day 7.5 and day 8.5, respectively) and more than mice immunized with either OMV preparation plus blank ms or sham-immunized mice (median clearance by day 11 in both cases) (Fig. 6A and B). Mice immunized with nOMVs plus IL-12 ms developed stronger serum IgG antibody responses than mice immunized with deOMVs plus IL-12 ms, and no significant levels of serum IgA antibodies were observed (Fig. 6C). In vaginal washes from mice immunized with nOMVs plus IL-12 ms, only IgA antibodies were detected, again at substantially higher levels than in deOMV-immunized mice (Fig. 6D). Flow cytometric analysis of iliac lymph node cells showed that mice immunized with either nOMVs or deOMVs plus IL-12 ms developed greater numbers of CD4^+^ cells producing IFN-γ than mice immunized with either preparation of OMVs plus blank ms or unimmunized control mice (Fig. 6E). There were no significant differences in the numbers of cells producing IL-4 between groups, and all mice generated IL-17A-secreting CD4^+^ cells as a result of infection, regardless of immunization (Fig. 6E).

Pooled serum from mice immunized with nOMVs or deOMVs (plus IL-12 ms) was assessed for the range of antigens identified in OMVs by means of Western blotting using nOMVs (20 μg protein) as target antigens separated by SDS-PAGE. As expected, deOMVs separated by SDS-PAGE revealed fewer protein bands than nOMVs, and four of these were detected by IgG antibodies in serum from mice immunized with deOMVs (Fig. 6F). In contrast, IgG antibodies in serum from mice immunized with nOMVs recognized at least seven protein bands, four of which were not present in deOMVs (Fig. 6F).

### OMVs from double mutant strain MS11.

**FIG 7:**
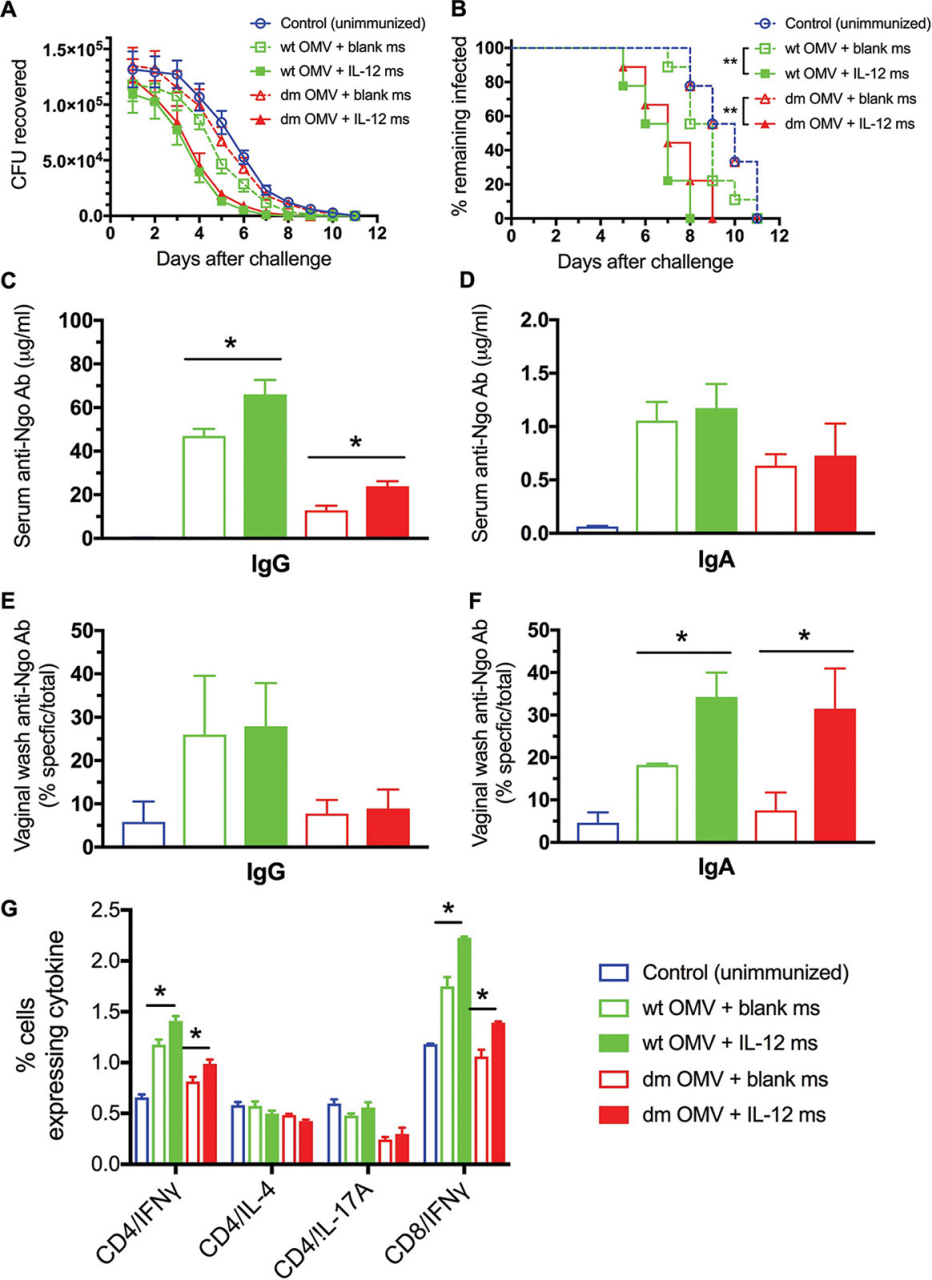
Infection of mice immunized i.n. with gonococcal OMVs from dm or wt MS11 plus blank ms or IL-12 ms with 5 × 10^6^ CFU of N. gonorrhoeae wt MS11. (A) Recovery of N. gonorrhoeae on vaginal swabs as each time point. Data are shown as means ± SEM (*n* = 9 mice per group). (B) Percentage of mice remaining infected at each time point. **, *P* < 0.002 (Kaplan-Meier log rank test). (C to F) Antigonococcal antibodies at termination for serum IgG (C), serum IgA (D), vaginal IgG (E), and vaginal IgA (F). (G) Intracellular cytokine staining in CD4^+^ T cells from iliac lymph nodes at termination. Data are shown as means ± SEM (*n* = 3 pools of 3 samples per group). *, *P* < 0.05 (Student’s *t* test).

A more precise way of reducing the endotoxicity of LOS is by eliminating one of the six acyl chains of lipid A, which can be achieved by genetically deleting the enzyme LpxL1 (24). In addition, we wanted to eliminate the outer membrane reduction-modifiable protein Rmp because this induces countereffective antibodies that block complement-mediated bacteriolysis as described previously (25). A double deletion mutant (dm) (Δ*lpxL1* Δ*rmp*) strain was constructed in N. gonorrhoeae strain MS11, and OMVs from this (dm OMVs) were compared with wild-type (wt) OMVs as vaccine immunogens by the i.n. route. Groups of 9 mice were immunized i.n. with dm or wt OMVs (30 μg protein), each with either IL-12 ms (1 μg IL-12) or blank ms, and a control group was left unimmunized. Immunizations were repeated at a 2-week interval, and mice were challenged by vaginal inoculation of live N. gonorrhoeae strain MS11. Mice immunized with dm OMVs plus IL-12 ms cleared the infection at the same rate as mice immunized with wt OMVs plus IL-12 ms (Fig. 7A). Statistical evaluation of time to clearance of infection by Kaplan-Meier analysis with log rank tests (Fig. 7B) showed no significant difference between immunization with dm and wt OMVs, with or without IL-12 ms (*P* > 0.9 in each case). Clearance was significantly faster in mice immunized with dm or wt OMVs plus IL-12 ms than in mice immunized with the same OMVs plus blank ms (Fig. 7B) or than in unimmunized control mice (*P* < 0.002 in each case). Immunization with dm or wt OMVs plus blank ms did not result in accelerated clearance relative to unimmunized mice (*P* > 0.9).

After clearance of the infections (~2 weeks after inoculation), samples of serum and vaginal wash were collected for antibody assay, and ILNs were harvested for analysis of cytokine production in T cells. Mice immunized with wt OMVs developed serum IgG antigonococcal antibody responses that were significantly increased by use of IL-12 ms, whereas mice immunized with dm OMVs developed much lower serum IgG antibodies, but these were also significantly increased by use of IL-12 ms (Fig. 7C). Serum IgA antibody responses were very low regardless of the immunizing OMVs or use of IL-12 ms (Fig. 7D). Vaginal IgG antibody responses appeared to reflect the serum IgG antibodies but did not attain significance (Fig. 7E), and vaginal IgA antibodies were significantly elevated in mice immunized with either dm or wt OMVs plus IL-12 ms (Fig. 7F). Higher numbers of IFN-γ-producing CD4^+^ T cells were found in ILNs of mice immunized with either wt or dm OMVs, and these were significantly increased by use of IL-12 ms (Fig. 7G). CD8^+^ IFN-γ-producing cells were also elevated in mice immunized with wt OMVs with or without IL-12 and in mice immunized with dm OMVs plus IL-12 ms (Fig. 7G). Production of IL-4 and IL-17 was not elevated in any immunized group (Fig. 7G).

## DISCUSSION

These results show that i.n. immunization with gonococcal OMVs plus IL-12 ms as adjuvant is at least as effective as i.vag. immunization with the same vaccine in mice, in terms of both the induction of antigonococcal antibody responses in serum and vaginal wash and in generating resistance to vaginal infection with N. gonorrhoeae. Interestingly, i.n. but not i.vag. immunization also induced salivary IgA antibodies as observed previously with different antigen and adjuvant combinations (20). Likely, this is because murine nasal passages contain nasopharyngeal-associated lymphoid tissue (21), which has a tissue architecture akin to intestinal Peyer’s patches and is able to function as an inductive site that disseminates stimulated lymphocytes throughout the mucosal immune system, including salivary glands as well as the genital tract (13, 28). In contrast, the genital tract does not contain inductive lymphoid tissue, typified by the presence of follicular T and B cell zones with an overlying follicle-associated epithelium containing M cells, and therefore is unable to disseminate responses to remote effector sites in the mucosal immune system (22, 29, 30). In addition, i.n. immunization with IL-12 ms induced the development of Th1 cells (i.e., IFN-γ-producing CD4^+^ cells in the iliac lymph nodes which drain the genital tract) in a similar manner to i.vag. immunization, at least after clearance of genital infection with N. gonorrhoeae. We previously showed that resistance to genital gonococcal infection depended on IFN-γ, as well as on B cells, presumably to generate antibodies (11, 31). Th2 (IL-4-producing CD4^+^) cells were not elevated by i.n. immunization with IL-12 ms, as noted previously for i.vag. immunization (11). Th17 (IL-17A-producing CD4^+^) cells were usually elevated after genital gonococcal infection regardless of immunization status, again as noted previously (10, 11, 31–35).

The success of i.n. immunization carries two important implications. First, it avoids potential difficulties arising from i.vag. immunization, which might include adverse impacts on reproductive tract function as well as acceptability for ultimate human application. Second, it is applicable to males as well as females. Here, we have shown that i.n. immunization of male mice induced similar serum and salivary antibody responses to those generated in female mice. Unfortunately, however, there is at present no known way of infecting male mice with N. gonorrhoeae to test for protection against genital infection, and we do not have the means to collect murine male genital secretions for antibody assay. Intranasal immunization has been shown to induce genital tract antibody responses in mice (12, 13, 19), monkeys (14), and koalas (36), as well as in humans, both male and female (15–17).

We previously reported that i.vag. immunization with gonococcal OMVs plus IL-12 ms induces antibodies that cross-react with antigenically different strains of N. gonorrhoeae and generates resistance to infection with heterologous strains (11). However, such studies were limited to three commonly available strains that have been extensively used in laboratory studies, although they retain virulence for humans, plus two uncharacterized clinical isolates. To expand the range of gonococcal strains tested, we obtained the 16 WHO gonococcal reference strains (23), which have been characterized for expression of the major outer membrane protein PorB as well as for resistance to antibiotics and expression of novel potential vaccine antigens (37). Remarkably, serum IgG antibodies induced in mice by i.n. immunization with OMV from either strain FA1090 (PorB1b) or strain FA19 (PorB1a) plus IL-12 ms cross-reacted in a screening assay with all 16 WHO reference strains regardless of PorB group. When three of these (after selection for resistance to streptomycin) were used in challenge infection studies of mice immunized i.n. with strain FA1090, resistance to infection against all three strains was similar to resistance to challenge with homologous FA1090. Moreover, serum and vaginal antibody levels against the challenge strains were comparable, implying equivalent recall responses against each strain. The three WHO strains tested include two that show increased resistance to antibiotics, including ciprofloxacin (L and W) as well as ceftriaxone (L) or cefixime (W) (23). While antibiotic resistance in itself is unlikely to be related to antigenic variability or immune escape, it is encouraging to note that a gonococcal OMV vaccine may be effective against antibiotic-resistant strains that are emerging as a major concern in the treatment of gonorrhea.

The nature of the antigens responsible for cross-protection between different gonococcal strains is unknown at present. Previously, in collaboration with Sikora et al., we identified two antigens (EF-Tu and PotF3) that were recognized by antibodies induced by i.vag. immunization with FA1090 OMVs (11). These antigens have also been identified in proteomics screening of multiple strains of N. gonorrhoeae (38), and likely more antigens would be identified by examining further samples of serum from i.n.- or i.vag.-immunized animals. Another possibility is that antibodies induced by OMV vaccine might recognize shared glycan determinants present on PilE and other surface proteins (39). In addition, LOS is an abundant surface molecule (26) present in the outer membrane, but it too is antigenically variable by means of phase variation of the glycosyltransferases that assemble the glycan chains (40, 41). As a polysaccharide, LOS is a T-independent antigen, which are typically less-effective immunogens than T-dependent antigens such as proteins, although one particular epitope, designated 2C7, has been the focus of attention as it is expressed by 95% of gonococcal isolates (42). However, gonococcal LOS is powerfully endotoxic and would be highly reactogenic in a vaccine, especially if administered parenterally. Although mucosal administration might be less problematic, there have been concerns over i.n. administration of a meningococcal OMV vaccine (43–45), which were addressed by detergent extraction to reduce the LOS content. This treatment, however, results also in extraction of some outer membrane proteins as well as diminished immunogenicity (26, 46). To address this potential problem, we compared the levels of immunogenicity and protective potential of detergent-extracted gonococcal OMVs with those of native OMVs from the same strain (FA19) prepared and provided by Intravacc. Although deOMVs given i.n. with IL-12 ms induced substantially lower serum and vaginal antibody responses as assessed by ELISA, with a different pattern of reactivity against OMV antigens separated by SDS-PAGE compared to nOMV vaccine, levels of resistance to genital gonococcal infection were similar with both OMV vaccines. This implies that efforts to diminish LOS toxicity in a gonococcal OMV vaccine might not necessarily result in diminished efficacy.

Somewhat similar results were obtained when mice were immunized i.n. with OMVs from dm mutant (Δ*lpxL1* Δ*rmp*) strain MS11, in that reduction of LOS endotoxicity and deletion of Rmp appeared to have no significant impact on the protective effect of the vaccine compared to wt OMVs when given i.n. with IL-12 ms. Both OMV preparations given with blank ms failed to induce significant resistance to challenge. However, the immune response in terms of antigonococcal antibody production in serum (IgG and IgA) and in vaginal wash (IgG) was diminished in mice immunized with dm OMVs compared to wt OMVs. Maintenance of vaginal IgA antibody production in mice immunized i.n. with dm OMVs plus IL-12 ms might simply reflect a mucosal antibody response, as distinct from a systemic antibody response that generated serum (and vaginal) IgG antibodies. IFN-γ-producing T cells were also diminished in mice immunized with dm OMVs, even when IL-12 ms was administered. These findings indicate that, despite the apparent reduction in immunogenicity of dm OMVs, generation of protective immunity was maintained in dm OMVs. In turn, this suggests that dm OMVs, which should have a lower reactogenicity profile as well as being incapable of inducing countereffective antibodies against Rmp, might be suitable for further development as a potential human vaccine.

Our findings, now demonstrated repeatedly in several studies on both therapeutic i.vag. treatment of infected mice with IL-12 ms and on immunization (i.n. or i.vag.) with gonococcal OMVs plus IL-12 ms (10, 11, 31), diverge from conventional thinking about (human) immune responses to N. gonorrhoeae, which is well known for its extraordinary antigenic variability. Thus, it has been held that while gonococcal infection (in humans) might induce strain-specific antibody responses, subsequent infection with a different strain would not meet with immune resistance because of antigenic differences in the new strain. Antigenic variation is well known as an immune evasion strategy exploited by many human pathogens, although few display it to the extent shown by N. gonorrhoeae. However, recent findings have cast doubt on this simple explanation. It now appears that N. gonorrhoeae interferes with the development of adaptive immune responses that might be effective against it, through several mechanisms, including inactivation of CD4^+^ T-helper cells (47), suppression of Th1/2-driven adaptive immune responses by upregulating transforming growth factor β (TGF-β), IL-10, and type 1 regulatory T cells (35), and modulation of antigen-presenting cell function (48, 49), as reviewed extensively in reference 8. In addition, a reduction of gonorrhea incidence has been retrospectively observed in several populations immunized with various meningococcal OMV vaccines (9, 50–54). OMVs from the New Zealand MeNZB vaccine are included in the recently licensed meningococcal vaccine Bexsero (GlaxoSmithKline), and subjects immunized with this vaccine developed antibodies that cross-react with N. gonorrhoeae (55, 56). Thus, it becomes plausible that immunity to gonococcal infection depends on responses to antigenic determinants that are shared not only between different strains of N. gonorrhoeae but also in the related species N. meningitidis. However, the degree of protection against gonorrhea induced by MeNZB vaccination was reported to be ~31% to 46%, and it declined with time elapsing after immunization (9, 54). Therefore, it is to be expected that a gonococcal OMV vaccine designed to induce immune responses against specific gonococcal antigens would be more effective.

It is noteworthy that, so far, we have not identified strains of N. gonorrhoeae that fail to react with antibodies induced against strain FA1090, MS11, or FA19 or that are not resisted when inoculated into mice immunized with vaccines prepared from these widely used strains. This is encouraging from the standpoint of achieving a vaccine that would be effective against most strains of N. gonorrhoeae. On the other hand, to find strains that are not immunologically cross-reactive with other common strains would provide valuable material for investigating the important target antigens of protective immunity. In this connection, we currently favor the view that protective immunity to N. gonorrhoeae is not necessarily dependent upon immune responses (presumably antibodies) to any particular target antigens, but on the sum total of antibodies against a sufficient range of antigens present on the gonococcal surface. On the other hand, studies reported here have begun to show some disconnection between antibody responses to immunization (as measured by ELISA against whole bacterial cells) and protection against gonococcal challenge. This suggests that antibody levels in serum or genital secretions might not be the key or sole determinants of protective immunity to N. gonorrhoeae. The functional properties of antibodies might be critical—for example, the abilities of different isotypes (including subclasses) to mediate antibacterial defense mechanisms, such as complement-dependent bacteriolysis, opsonophagocytosis, or inhibition of attachment to and invasion of epithelial cells. Other mechanisms can be envisioned, including the role of IFN-γ, a pleiotropic and multifunctional cytokine, which we have found to be important in mice (11, 31), but in the absence of known determinants or even correlates of gonococcal immunity in humans, these all remain to be investigated.

## MATERIALS AND METHODS

### Mice.

BALB/c mice 6 to 8 weeks of age were purchased from Jackson Laboratories (Bar Harbor, ME) and were maintained in a biosafety level 2 suite in the Laboratory Animal Facility at the University at Buffalo, which is fully accredited by AAALAC. All animal use protocols were approved by the Institutional Animal Care and Use Committee of the University at Buffalo.

### Bacteria.

N. gonorrhoeae strains FA1090, MS11, and FA19 were obtained as described previously (11). WHO reference strains of N. gonorrhoeae (23) were kindly provided by Aleksandra Sikora (Oregon State University). All strains were cultured on GC agar supplemented with hemoglobin and Isovitalex (BD Diagnostic Systems, Franklin Lakes, NJ) incubated at 37°C under 5% CO_2_–air, and the resultant growth was checked for colony morphology consistent with Opa protein and pilus expression. Bacteria were harvested from plates, and the cell density was determined as detailed previously (34).

For use in genital infection of mice, WHO strains were rendered streptomycin resistant (27) by being inoculated heavily on multiple GC agar plates containing streptomycin at 100 μg/mL and incubated for 2 to 3 days. Three strains (WHO F, L, and W) that showed moderate cross-reactivity with serum from mice immunized with FA1090 or FA19 (see Table S1 in the supplemental material) yielded colonies that grew in the presence of streptomycin, and these were expanded for use in challenge infection experiments.

To generate the *lpxL1* and *rmp* deletion mutations in strain MS11, a spontaneous streptomycin-resistant colony was isolated. Marker-free mutagenesis was performed on this strain as described previously (57) to delete *lpxL1* and *rmp* genes. Deletions were verified by PCR.

### Gonococcal outer membrane vesicles.

Three preparations of OMVs from gonococcal strains FA1090, MS11, or FA19 were used in this study, obtained by extraction in lithium acetate, deoxycholate, or EDTA. The lithium acetate-extracted nOMVs (LiAcOMVs) were prepared in the Therapyx laboratory by sheering gonococci (FA1090) harvested from supplemented GC agar plates in lithium acetate buffer followed by ultracentrifugation as described previously (11). Protein was assayed with the Micro BCA (bicinchoninic acid) protein kit (Thermo Scientific, Rockford, IL) or RC DC protein assay kit (Bio-Rad, Hercules, CA).

Detergent (deoxycholate)-extracted OMVs (deOMVs) were isolated from strain FA19 in the Intravacc laboratories after growth in bioreactors as described previously (58), with the following modifications: the CO_2_ level was held constant at 5% and the pH was set at 6.3. After cultivation, the deOMVs were isolated as described previously (59). EDTA-extracted nOMVs and the double mutant (Δ*lpxL1* and Δ*rmp*) MS11 (dm OMVs) were isolated after growth in bioreactors as described above for deOMVs using a mild EDTA extraction procedure instead of deoxycholate (60).

### IL-12 microspheres.

Murine IL-12 (Wyeth, Philadelphia, PA) was encapsulated into polylactic acid microspheres by phase inversion nanoencapsulation technology as previously described, except that bovine serum albumin was replaced with sucrose (0.1% [wt/wt]) (61). Blank microspheres were prepared in the same way but without IL-12.

### Intravaginal and intranasal immunization.

For i.vag. immunization, groups of 8 or 9 female mice between 7 and 9 weeks old were immunized with gonococcal OMVs (30 μg protein) plus IL-12 ms (1 μg IL-12) or blank ms in a total volume of 40 μL phosphate-buffered saline (PBS) as described previously (11). For i.n. immunization, gonococcal OMVs (30 μg total protein) plus IL-12 ms (1 μg IL-12) or blank ms were suspended in a total volume of 20 μL PBS and gently instilled into the external nares (5 to 10 μL per nostril at a time) using a pipettor fitted with a fine tip. Immunizations were repeated once after 14 days. In some experiments, control groups were sham immunized with PBS alone.

### Vaginal infection.

Approximately 3 to 4 weeks after the last immunization dose, mice were infected with live N. gonorrhoeae as previously described (27, 35), with the modification that 0.5 mg Premarin (Pfizer, Philadelphia, PA) was used as estradiol administered subcutaneously (s.c.) on days −2, 0, and 2. Vaginal swabs collected daily were quantitatively diluted and cultured on GC agar supplemented with hemoglobin, Isovitalex, and selective antibiotics (vancomycin, streptomycin, nisin, colistin, and trimethoprim) for 16 to 24 h at 37°C under 5% CO_2_–air. Plates were counted without reference to the experimental condition to determine the bacterial colonization loads (27, 35). The limit of detection was 100 CFU recovered per mouse.

### Assay of serum and mucosal antibodies.

Samples of serum, vaginal wash, and saliva were collected from the mice 2 weeks after the last immunization, or at termination of the experiment after clearance of the infection (~2 weeks after inoculation) (10, 33). Gonococcus-specific IgG and IgA antibodies in serum and vaginal washes were assayed by ELISA on plates coated with whole gonococci of the strain used for vaginal challenge unless stated otherwise (10, 33). Total IgA and IgG concentrations in secretions were assayed by ELISA on plates coated with anti-IgA or anti-IgG antibodies (Southern Biotech, Birmingham, AL). H5 mouse monoclonal antibody (specific for N. gonorrhoeae porin serovar PIB3 in FA1090) was used to establish standard curves when IgG antibodies were being assayed against FA1090. Otherwise, affinity-purified mouse IgG or IgA (Southern Biotech) was used to establish standard curves on plates coated with anti-mouse IgG or IgA, respectively. Bound antibodies were detected by alkaline phosphatase-conjugated goat anti-mouse IgG or IgA antibody (Southern Biotech) and *p*-nitrophenyl phosphate substrate (Southern Biotech). Plates were read in an EL_X_800 Universal microplate reader with Gen 5 software (Bio-Tek Instruments, Winooski, VT).

### Lymphocyte staining for cytokines.

Cells recovered from iliac lymph nodes (ILNs) were washed with staining buffer twice and then incubated with the indicated antibodies for 30 min on ice, washed, and analyzed on a FACSCalibur cytometer in the Optical Imaging and Analysis Facility, University at Buffalo School of Dental Medicine. For intracellular staining, cells were first fixed with Cytofix/Cytoperm (eBioscience). Antibodies to mouse CD4, IFN-γ, IL-4, and IL-17A conjugated with fluorescein isothiocyanate (FITC), phycoerythrin (PE), or allophycocyanin were purchased from eBioscience.

### Western blotting.

Gonococcal OMV preparations were boiled for 5 min in SDS loading buffer containing 2-mercaptoethanol. Protein quantification was done with the RC DC protein assay kit. Twenty micrograms of protein from each sample was separated on 10% polyacrylamide–SDS electrophoresis gels. Protein bands were transferred onto nitrocellulose membranes using the electrophoresis transfer system (Bio-Rad, Hercules, CA, USA). The membranes were blocked with PBS containing 3% skim milk overnight at 4°C before incubation for 2 h with serum samples diluted 1:200 or vaginal wash samples diluted 1:20 in PBS containing 3% skim milk. Specific antibodies bound to N. gonorrhoeae OMV preparations were detected with horseradish peroxidase-conjugated goat anti-mouse IgG (Santa Cruz Biotechnology, Paso Robles, CA) at a dilution of 1:4,000. The Pierce detection kit was used for chemiluminescent detection, and images were collected with a ChemiDoc MP imaging system (Bio-Rad). Replicate gels were stained using Coomassie blue to reveal protein bands.

### Statistical analysis.

Data on the effect of immunization on recovery of N. gonorrhoeae after inoculation were analyzed using two-way ANOVA for repeated measures with Tukey’s multiple comparisons. In addition, Kaplan-Meier survival analysis with Mantel-Cox log rank tests was used to compare levels of clearance of infection (defined as the first of 3 successive days of zero recovery) between treatment groups. For immune response data, unpaired two-tailed Student’s *t* tests or one-way ANOVA with Tukey’s multiple comparisons were used as appropriate. A *P* value of <0.05 was considered statistically significant. Statistical analyses were performed using Prism 7 (GraphPad Software, San Diego, CA).
