# Supplementary material for: Microencapsulated IL-12 Drives Genital Tract Immune Responses to Intranasal Gonococcal Outer Membrane Vesicle Vaccine and Induces Resistance to Vaginal Infection with Diverse Strains of Neisseria gonorrhoeae
Source: mSphere. 2022 Dec 20;8(1):e00388-22. doi: 10.1128/msphere.00388-22 (PMC9942569; doi:10.1128/msphere.00388-22)
Supplement: FIG S1 [file msphere.00388-22-s0002.pdf]

Supplementary Fig. 1

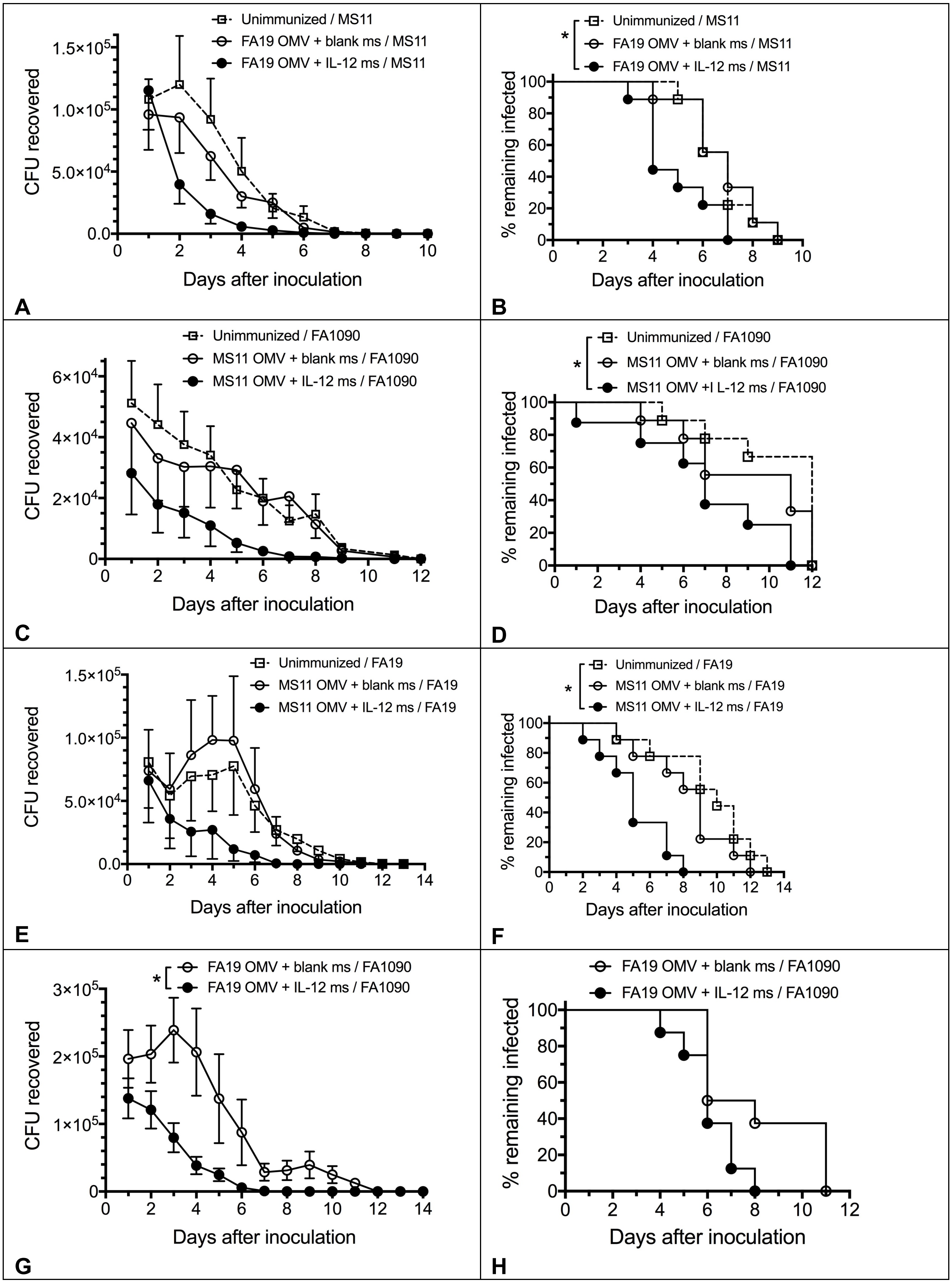

Supplementary Fig. 1: Cross-protection against heterologous strains of *N. gonorrhoeae* induced by i.n. immunization with various strains of gonococcal LiAcOMV plus IL-12 ms.
A, B: Immunization with FA19 LiAcOMV plus IL-12 ms or blank ms, challenged with MS11 (N=9). * P =0.0230 (Kaplan-Meier, log-rank test).
C, D: Immunization with MS11 LiAcOMV plus IL-12 ms or blank ms, challenged with FA1090 (N=8/9). * P <0.01 (Kaplan-Meier, log-rank test).
E, F: Immunization with MS11 LiAcOMV plus IL-12 ms or blank ms, challenged with FA19 (N=9). * P =0.0120 (Kaplan-Meier, log-rank test).
G, H: Immunization with FA19 LiAcOMV plus IL-12 ms or blank ms, challenged with FA1090 (N=9). * P <0.02 (2-way ANOVA).
