# Supplementary material for: Microencapsulated IL-12 Drives Genital Tract Immune Responses to Intranasal Gonococcal Outer Membrane Vesicle Vaccine and Induces Resistance to Vaginal Infection with Diverse Strains of Neisseria gonorrhoeae
Source: mSphere. 2022 Dec 20;8(1):e00388-22. doi: 10.1128/msphere.00388-22 (PMC9942569; doi:10.1128/msphere.00388-22)
Supplement: TABLE S1 [file msphere.00388-22-s0001.pdf]

Supplementary Table 1: Cross-reactivity of sera from mice immunized i.n. with gonococcal OMVs against *N. gonorrhoeae* WHO reference strains. Results shown as % of IgG reactivity against each strain relative to reactivity against homologous immunizing strain (shown as 100%). Figures in **bold** are >50%.

Immun-ization strain (OMV)	Reactivity against *N. gonorrhoeae* strain (porin type shown below each)															
	FA19	FA1090	WHO F	WHO G	WHO K	WHO L	WHO M	WHO N	WHO O	WHO P	WHO U	WHO V	WHO W	WHO X	WHO Y	WHO Z
	PorB1a	PorB1b	PorB1a	PorB1a	PorB1b	PorB1b	PorB1b	PorB1a	PorB1b	PorB1b	PorB1b	PorB1b	PorB1b	PorB1b	PorB1b	PorB1b
FA19	**100**	41	32	40	**69**	**54**	42	43	40	48	**63**	45	**50**	36	33	**71**
FA1090	NT	**100**	45	40	**70**	**59**	43	40	**62**	25	35	**57**	49	23	42	37

NT: not tested
